# Effects of Age and Acute Ethanol on Glutamatergic Neurotransmission in the Medial Prefrontal Cortex of Freely Moving Rats Using Enzyme-Based Microelectrode Amperometry

**DOI:** 10.1371/journal.pone.0125567

**Published:** 2015-04-30

**Authors:** Devesh Mishra, Nicholas R. Harrison, Carolina B. Gonzales, Björn Schilström, Åsa Konradsson-Geuken

**Affiliations:** 1 Department of Physiology and Pharmacology, Karolinska Institutet, Stockholm, Sweden; 2 Department of Neuroscience and Physiology, Section of Neuropsychiatry, Gothenburg University, Gothenburg, Sweden; 3 Department of Neuroscience, Karolinska Institutet, Stockholm, Sweden; Radboud University, NETHERLANDS

## Abstract

Ethanol abuse during adolescence may significantly alter development of the prefrontal cortex which continues to undergo structural remodeling into adulthood. Glutamatergic neurotransmission plays an important role during these brain maturation processes and is modulated by ethanol. In this study, we investigated glutamate dynamics in the medial prefrontal cortex of freely moving rats, using enzyme-based microelectrode amperometry. We analyzed the effects of an intraperitoneal ethanol injection (1 g/kg) on cortical glutamate levels in adolescent and adult rats. Notably, basal glutamate levels decreased with age and these levels were found to be significantly different between postnatal day (PND) 28-38 vs PND 44-55 (p<0.05) and PND 28-38 vs adult animals (p<0.001). We also observed spontaneous glutamate release (transients) throughout the recordings. The frequency of transients (per hour) was significantly higher in adolescent rats (PND 28-38 and PND 44-55) compared to those of adults. In adolescent rats, post-ethanol injection, the frequency of glutamate transients decreased within the first hour (p<0.05), it recovered slowly and in the third hour there was a significant rebound increase of the frequency (p<0.05). Our data demonstrate age-dependent differences in extracellular glutamate levels in the medial prefrontal cortex and suggest that acute ethanol injections have both inhibitory and excitatory effects in adolescent rats. These effects of ethanol on the prefrontal cortex may disturb its maturation and possibly limiting individuals´ control over addictive behaviors.

## Introduction

Brain development and maturation continues during adolescence until adulthood in humans [1]. There are continuous morphological and functional alterations within the brain, modulated by external psychosocial, environmental, cultural, economic and biological factors that shape the individuals´ behavior as an adult [2]. Synaptic pruning is one such remodeling mechanism occurring in the prefrontal cortex during adolescence, leading to dramatic changes in the number of synapses [2, 3]. Excitatory glutamatergic *N*-methyl-D-aspartate (NMDA) receptors play a critical role in processes involved in synapse strengthening or removal [4–7]. Therefore, the excitatory neurotransmitter glutamate, activating the NMDA receptor, likely plays an important role in normal brain development. Ethanol consumption during adolescence has been suggested to interfere with maturational processes and can have detrimental effects on overall brain development. In particular, the development of the prefrontal cortex which, among other functions, imparts an inhibitory control over behaviors like risk-taking and ethanol over-consumption may be drastically impaired and may predispose the individual (adolescent) to the risk of developing ethanol addiction later in life [8, 9]. Even so, we know surprisingly little about glutamatergic neurotransmission in the prefrontal cortex during development and how it is affected by ethanol. A previous study using adult animals showed that ethanol had no effect on glutamate levels when measured by microdialysis [10]. However, ethanol has been shown to inhibit firing activity of neurons in the prefrontal cortex, an effect that could be mimicked by the NMDA receptor antagonist APV [11]. Using electrophysiological recordings in slice preparations from pre-adolescent animals, acute exposure to ethanol has also been shown to inhibit NMDA receptor-mediated excitatory postsynaptic currents in the deep layer pyramidal neurons of the prefrontal cortex, suggesting no effect on glutamate release [12]. Ethanol may, therefore, exert an inhibitory effect on glutamatergic neurotransmission mainly via blockade of postsynaptic NMDA receptors.

Nevertheless, along with the well described inhibition of postsynaptic NMDA receptors, several electrophysiological studies in various brain areas using slices taken from adolescent animals suggest that ethanol may also inhibit glutamate release [13–16] and thereby indicate that the inhibitory effect of ethanol on glutamatergic transmission may be both pre- and postsynaptic, depending on age and brain region studied. In the present study, we used an enzyme-based microelectrode array (MEA) in freely moving animals with which both tonic and phasic (i.e. transients) glutamate release and clearance can be investigated with better temporal and spatial resolution than microdialysis (for details, see [17–20]). We hypothesized that basal glutamate levels in the medial prefrontal cortex (mPFC) may differ with age and, that acute administration of ethanol would have an inhibitory effect on glutamate release in the mPFC, and that the level of inhibition might differ between adolescent and adults.

## Materials and Methods

### Animals

Male Sprague Dawley rats (Charles River, Germany) were used in our experiments. Rats were divided into the following age groups: postnatal day (PND) 28–38, PND 44–55 and adult animals (3–5 months old) [21, 22]. Animals were maintained in a temperature and humidity controlled facility on a 12:12-h light/dark cycle (lights on at 6 AM) and the animals had free access to food and water. All animals were housed in the same room with 5 animals in each cage handled for a total of 5 days and habituated to saline intraperitoneal (i.p.) injections, 6 ml/kg/day for 3 consecutive days before surgery.

### Ethical Statement

All efforts were made to minimize animal suffering and to reduce the number of animals used throughout the project. The experiments were approved by Stockholms Norra Djurförsöksetiska nämnd, ethical permit numbers: N602/12, N457/12 and N319/12.

### Materials

L-ascorbic acid (AA), dopamine, L-glutamate (monosodium salt), glutaraldehyde [25% (w/v) in water], bovine serum albumin (BSA), *m*-phenylenediamine dihydrochloride (*m*-PD) and hydrogen peroxide (H_2_O_2_) were ordered from Sigma Aldrich Corp. (St. Louis, MO, USA). Sodium chloride (NaCl) was purchased from VWR Leuven, Belgium. L-Glutamate oxidase (GluOx; EC 1.4.3.11) was purchased from Heamochrom Diagnostics AB, ethanol (95%) was purchased from Solveco AB, sodium barbiturate was bought from Apoteket AB and isoflurane was purchased from Baxter Medical AB, all companies based in Sweden. Ethanol was dissolved in physiological saline for i.p. injections. Other solutions were prepared using distilled and deionized water.

### Detecting glutamate signals in freely moving animals using enzyme based microelectrode array (MEA)

**Fig 1:**
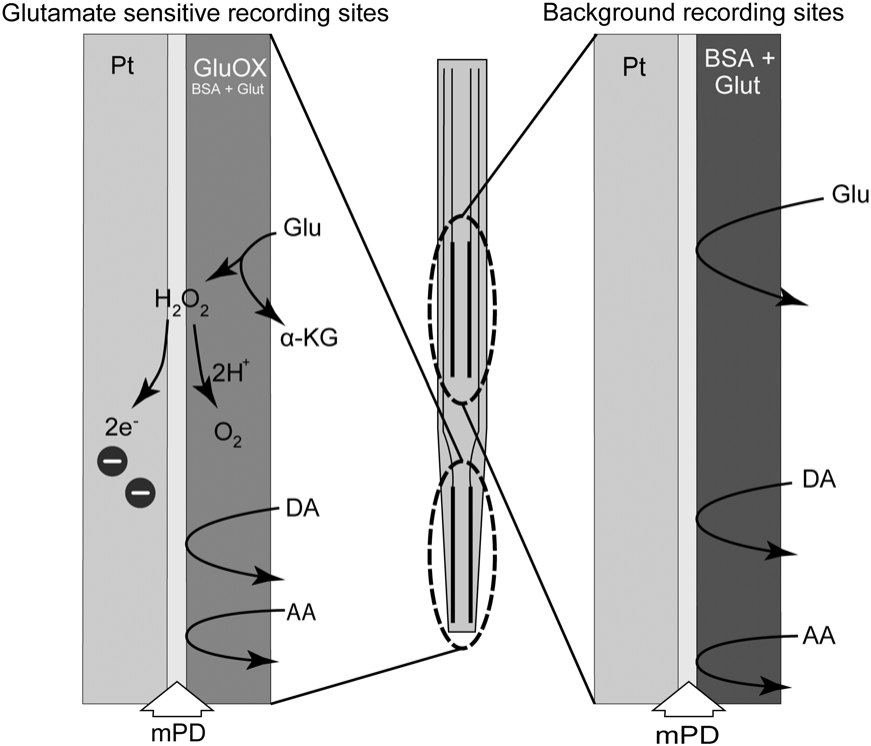
The enzyme scheme used in the detection of glutamate. The tip of the microelectrode consists of two pairs of platinum recording sites. One pair, the glutamate sensitive sites, were coated with a mixture of glutamate oxidase (GluOx), bovine serum albumin (BSA) and glutaraldehyde (0.125%). The remaining pair was coated only with BSA and glutaraldehyde and they served as control (background/sentinel) channels sensitive to the oxidation of endogenous molecules other than glutamate. *m*-phenylenediamine dihydrochloride (*m*-PD) was electropolymerized onto all sites of the microelectrode in order to reduce access of potential electroactive interferents, like ascorbic acid (AA) and catecholamines, to the platinum recording sites (Mitchell, 2004). Released glutamate is oxidized by GluOx at the glutamate-sensitive sites, generating α-ketoglutarate and H_2_O_2_. Since the microelectrode is maintained at a constant potential (+0.7 V versus an Ag/AgCl reference), the H_2_O_2_ reporting molecule is further oxidized, yielding two electrons. The resulting current is then amplified and recorded by a FAST-16 recording system (Quanteon, LLC, Nicholasville, KY, USA). Extracellular glutamate reaches the platinum surface of control sentinels (without GluOx) but no oxidation current is generated. Therefore, any current detected at these sites is due to electrochemically active interferents other than glutamate.

The microelectrode consists of a ceramic paddle with four platinum recording sites, for details about structure and assembly of the microelectrodes; see Konradsson-Geuken *et al*. [19]. The sites are arranged in two pairs beginning approximately 100 μm from the electrode tip. To design a glutamate sensitive microelectrode, one pair of recording site was coated with a mixture of GluOx (2%, 0.5 unit/1 μL), BSA (1%) and glutaraldehyde (0.125%). The remaining pair was coated only with BSA (1%) and glutaraldehyde (0.125%) to serve as control (sentinel/background) channels, sensitive to the oxidation of all endogenous molecules other than glutamate. As shown in Fig 1, this selective coating of the microelectrode allows for a self-referenced recording in which the current derived exclusively from glutamate oxidation can be isolated [17–20]. Enzyme-coated microelectrodes were allowed to dry for at least 48 hours at room temperature before any further use. Briefly, the released glutamate is oxidized by GluOx at the glutamate-sensitive sites, generating α-ketoglutarate and H_2_O_2_. Since the microelectrode is maintained at a constant potential (+0.7 V versus an Ag/AgCl reference electrode), the H_2_O_2_ reporting molecule is further oxidized, yielding two electrons (see Fig 1). The resulting current is then amplified and recorded by a FAST-16 recording system (Quanteon, LLC, Nicholasville, KY, USA). Extracellular glutamate reaches the platinum surface of control sentinels (without GluOx), but no oxidation current is generated. Therefore, any current detected at these sites is due to electrochemically active molecules other than glutamate.

### *In vitro* calibration

**Fig 2:**
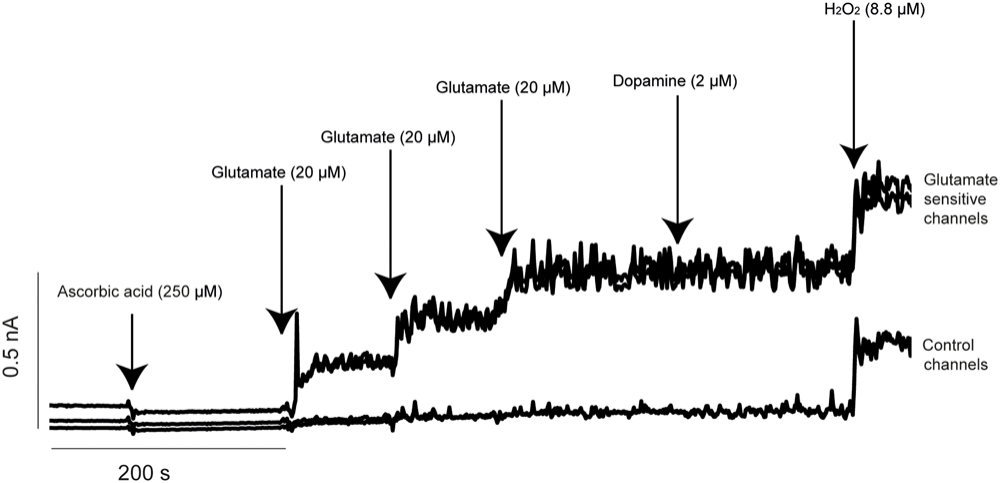
A representative *in vitro* calibration of the microelectrode performed prior to implantation into the mPFC. The top two tracings represent recording from glutamate-sensitive channels (coated with glutamate oxidase) and the bottom two tracings are generated from control channels. Arrows represent the addition of various substances into the calibration beaker. Current (in nA) is shown along the vertical axis and time in seconds along the horizontal axis. Three successive additions of glutamate (20 μM/aliquot) produced a linear increase of current on the glutamate sensitive channels. Expectedly, there were no changes in current detected on the two control channels. The calibration also shows comparable sensitivities on all four channels to the reporting molecule H_2_O_2_. *m*-PD was efficient in blocking the changes in currents due to potential electroactive interferents, i.e. ascorbic acid (AA) and dopamine (DA).

Prior to calibration *m*-PD (5.0 mM) was electropolymerized onto all sites of the microelectrode in order to reduce access of potential electroactive interferents, like AA and catecholamines, to the platinum recording sites [21]. The electroplating was done in nitrogen bubbled phosphate-buffered saline 0.05 M, using the FAST-16 electroplating tool (peak-to-peak amplitude of 0.25 V every 0.05 s for 22 minutes). Microelectrodes were calibrated *in vitro* immediately prior to implantation. Calibrations were performed in a stirred solution of phosphate-buffered saline (0.05 M, 40 mL, pH 7.4, 37°C). A stable baseline was established, AA (250 μM), three aliquots of glutamate (20 mM; resulting in to a final concentration of 20, 40 and 60 μM), dopamine (2 μM), and H_2_O_2_ (8.8 μM) were sequentially added to the calibration beaker (see Fig 2). Amperometric signals were acquired at a rate of 2.0 Hz. The sensitivity (pA/μM glutamate), limit of detection (LOD) for μM glutamate concentration (i.e. the smallest signal in glutamate concentration detected), selectivity (ratio of glutamate over AA), and linearity (R^2^) were calculated. The microelectrodes to be used for implantation and further *in vivo* recordings had to fulfill the following calibration criteria: (i) similar background current (i.e. less than 20 pA difference between the glutamate-sensitive and control sentinel channels), (ii) linear response to increasing concentrations of glutamate (R^2^ close to 1), (iii) a minimum glutamate sensitivity of -0.003 nA/μM glutamate, (iv) a LOD of ≤0.5 μM, and (v) a high selectivity for glutamate over AA and dopamine (i.e. >50:1).

### *In vitro* calibration in presence of ethanol

Following an injection of 1 g/kg ethanol the blood and brain concentrations of ethanol are comparable and expected to be approximately 20 mM within the first hour post injection [22]. To make sure that this ethanol concentration did not affect the sensitivity of the glutamate microelectrode (i.e. the activity of the enzyme), three different *in vitro* calibrations with ethanol added to the beaker was performed; i) a full calibration as described above ii) a full calibration with addition of 20 mM ethanol in the end of the calibration, and finally iii) a full calibration in 20 mM ethanol. Ethanol in a concentration of 20 mM did not affect the sensitivity (remained unchanged: 0.005 nA/μM) of the microelectrode in any of the calibrations. Furthermore, the LOD and the linear response to increasing concentrations of glutamate were also unaffected (LOD: 0.233 μM vs 0.303 μM in both calibrations, linearity to increasing glutamate concentrations: 1.0). Finally, ethanol did not affect the recorded current (nA) when added at the end of the calibration.

### Surgery and implantation of microelectrode

Surgery was performed under continuous anesthesia (isoflurane with air (30% O_2_ and 70% N_2_,1–3 L/min, 1–3% v/v). A microelectrode was unilaterally implanted in the mPFC in PND 28–38 at AP: +3.0 from bregma, ML: ± 0.8 mm from midline, and DV:- 5.3 mm from dura; PND 44–55 at AP: + 3.1 mm from bregma, ML: ± 0.9 mm from midline, and DV:- 5.8 mm from dura [23] and in adults AP: + 2.7 mm from bregma, ML: ± 0.6 mm from midline, DV:- 3.9 mm from dura. The stereotaxic coordinates for adults were determined using Paxinos and Watson brain atlas, fourth edition [24]. An Ag/AgCl reference electrode was implanted on the contralateral side inside a brain region distant from the recording area. Following surgery, every animal was individually housed and allowed to recover for 48 hours before any experimental recordings. The recovery of the animals was monitored at least once per day post-surgery.

### *In vivo* recordings with acute intraperitoneal injection

**Fig 3:**
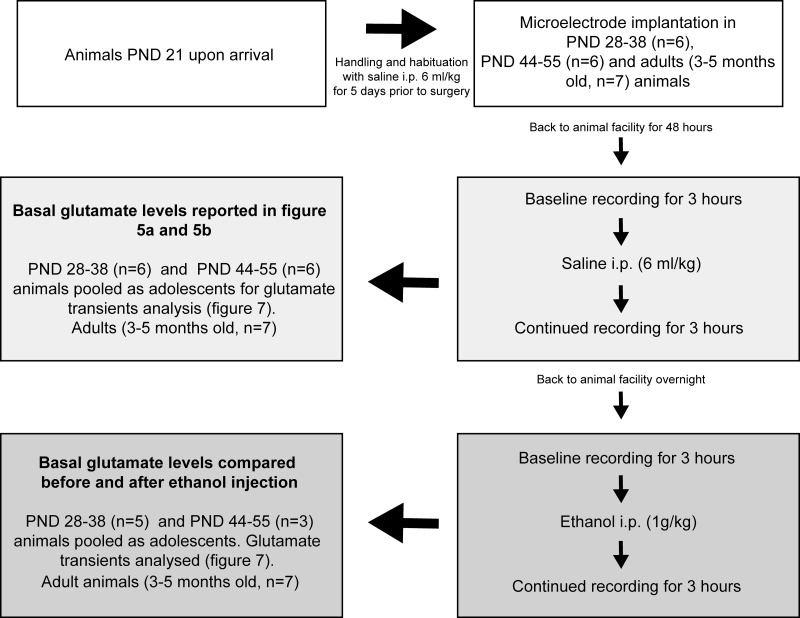
Experimental design. The number of animals in each age group, the experimental days, data collection and how age groups were pooled for statistical analysis are provided above. PND: postnatal day, i.p: intraperitoneal injection.

Recordings were conducted during day time between 6 AM and 6 PM in freely moving rats in a wooden box (H: 55 cm, W: 51 cm, L: 55 cm). Animals were placed in the recording box and connected to a head stage. On the saline day (experimental day 1) of recording, stable baseline signals were recorded for 3–4 hours followed by an i.p. injection of physiological saline (6 ml/kg), the recording was then continued for an additional three hours. Following experimental day 1 recordings, the animal was taken back to the animal facility. The next day the same procedure and time schedule was repeated (experimental day 2) with an i.p. injection of ethanol (1 g/kg, 6 ml/kg volume) to study the ethanol specific effects on glutamate dynamics. For details on the experimental design, see Fig 3.

### Histological verification of microelectrode placement

**Fig 4:**
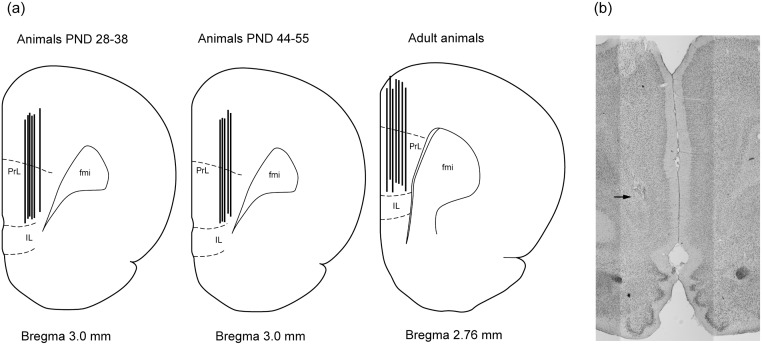
Localization of the microelectrode in the mPFC. a) Localization of microelectrodes in the mPFC of animals aged PND 28–38, PND 44–55 and adult animals according to Paxinos and Watson brain atlas, fourth edition [24]. PL: prelimbic, IL: infralimbic, fmi: forceps minor. b) Photomicrograph illustrating a representative placement of the microelectrode in the mPFC of a PND38 rat, (coronal section). The end of the platinum tip of the microelectrode (i.e. the recording sites) is indicated by the arrow.

In the end of experimental day 2, the animals were sacrificed by a lethal dose of sodium barbiturate followed by decapitation. Brains were removed and stored in 25% sucrose 4% formaldehyde solution at 4°C for at least 24 hours. Coronal brain sections of 50 μm were cut using a cryostat and mounted on gelatin-coated slides, stained using neutral red and examined under a light microscope for verification of microelectrode placement. The region of interest and the localization of microelectrodes implanted in the mPFC of all age groups are shown in a schematic picture (Fig 4a). Fig 4b illustrates a representative position of microelectrode in a coronal mPFC section from a PND 38 animal.

### Data analysis

The glutamate signal, initially measured in current (pA), was converted to a concentration equivalent (μM) with FAST analysis software version 4.4, on the basis of the individual calibration curves generated immediately before surgery. The basal levels were calculated based on the difference between glutamate sensitive and control sentinel as previously described [17, 18, 25, 26]. Glutamate levels were reported as an average of 15 minutes of recordings during baseline before injection and the first 15 min from each first, second and third hour following injection. Glutamate transients were sorted using control R^2^ (metric correlation between the glutamate and control channel) values. A high R^2^ (close to 1) indicates that glutamate and control sites are correlated and the signal is not electrochemical (due to glutamate), but rather noise. So only the peaks with R^2^ values <0.55 were selected for further analysis. Peak threshold value was set to 3 times the baseline noise values for all the recordings. In some animals (n = 1 in the PND 28–38 group, n = 3 in PND 44–55 group) the recordings had to be discontinued as the microelectrode complex was detached from the skull during injection of the animals. Data recorded before the detachment of the microelectrode complex has been used for calculating the basal levels of glutamate. Data was evaluated for significance using two-way ANOVA followed by Bonferroni’s post-hoc tests for multiple comparisons or the two-tailed Student’s t-test for paired and unpaired observations using GraphPad Prism version 6.

## Results

Before surgery and implantation of the microelectrodes, *in vitro* calibrations were performed. The obtained calibration values are presented in Table 1.

**Table 1:** Values from *in vitro* calibrations of microelectrodes prior to implantation.

Sensor Implanted	Sensitivity (nA/μM)	Linearity (R^2^)	Selectivity (glutamate/AA)	LOD
In Adults	-0.0087 ± 0.0011	0.9991 ± 0.0001	225 ± 57.1	0.27 ± 0.07
In PND 28–38	-0.0060 ± 0.0027	0.9990 ± 0.0004	259 ± 77.1	0.34 ± 0.05
In PND 44–55	-0.0061 ± 0.0013	0.9992 ± 0.0003	199 ± 95.1	0.42 ± 0.09

### Basal glutamate levels

**Fig 5:**
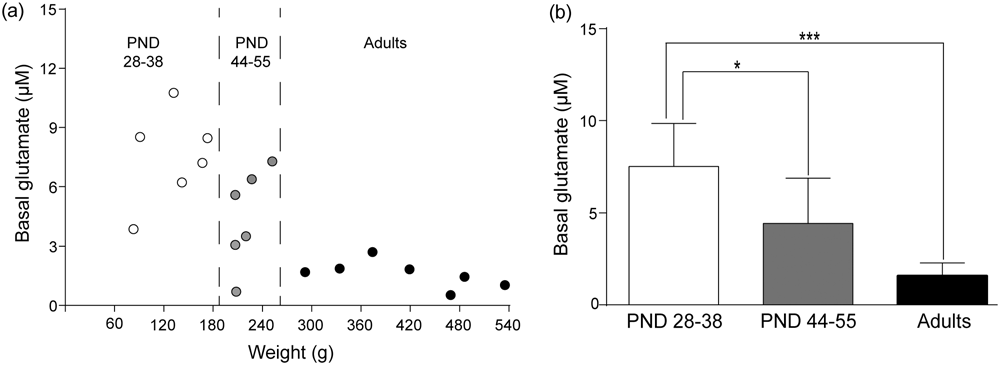
Basal glutamate on experimental day 1 a) Basal glutamate levels expressed as a function of age range and their corresponding weights on the day of recording. Each circle represents an individual animal. b) The cortical levels (μM) of glutamate in animals in postnatal day (PND) 28–38 were more than three times higher compared to that of adults (3–5 months old). Glutamate levels decreased with increasing age. Significance was tested using two-way ANOVA followed by Bonferroni´s post-hoc comparison test. *p<0.05, ***p<0.001.

On experimental day 1, glutamate levels were determined before and after saline injections. Baseline glutamate levels decreased with increasing age (see Fig 5a and 5b). The basal glutamate level in 28–38 day old animals was 7.5 μM ± 1.0 (n = 6) while the basal value in animals aged 44–55 days was 4.4 μM ± 1.0 (n = 6). The basal glutamate level in adult animals was 1.7 μM ± 0.3 (n = 7). Following saline injection glutamate levels were, 7.7 μM ± 1.1 (n = 6) in PND 28–38, 3.8 μM ± 1 (n = 6) in PND 44–55 and 1.5 μM ± 0.3) (n = 7) in adult animals. A two-way ANOVA revealed an effect of age (F_2,16_ = 15.06, p<0.001), but not of saline injection (F_1,16_ = 0.68, p>0.05) and there was no interaction effect over time (F_2,16_ = 0.92, p>0.05). Post hoc analysis using Bonferroni’s multiple comparisons test showed following significant differences between basal glutamate levels of the different age groups: i) PND 28–38 vs PND 44–55 (p<0.05) and adults vs PND 28–38, (p<0.001), see Fig 5b).

On experimental day 2, the basal glutamate level in the PND 28–38, PND 44–55 and adult animals were 11.3 μM ± 3.2, 7.1 μM ± 2.8 and 1.2 μM ± 0.2, respectively. When compared with basal glutamate levels on day 1, we did not observe any significant differences. The statistical values are as follows PND 28–38 (day 1 vs day 2, n = 6, p>0.5, t_5_ = 0.71), PND 44–55 (day 1 vs day 2, n = 6, p>0.3, t_5_ = 0.97) and in adult animals (day 1 vs day 2, n = 7, p>0.2, t_5_ = 1.25).

### Glutamate levels following ethanol injection

Following ethanol there was a slight decrease in glutamate levels in the PND 44–55 group from 7.1 μM ± 2.8 to 6.4 μM ± 2.9 during the third hour. Therefore, statistical analysis was performed on glutamate levels during this time point. A two-way ANOVA again revealed an age-difference (F_2,14_ = 4.20, *p<0.05, see above), but no effect of ethanol (F_1,14_ = 1.64, p>0.05) and no interaction effect over time (F_2,14_ = 1.11, p>0.05). In the third hour after acute ethanol injection glutamate levels in PND 28–38 and adult animals were 11.9 μM ± 3.7 (n = 5) and 1.0 μM ± 0.2 (n = 7), respectively.

### Glutamate transients and effects of saline or ethanol injection

**Fig 6:**
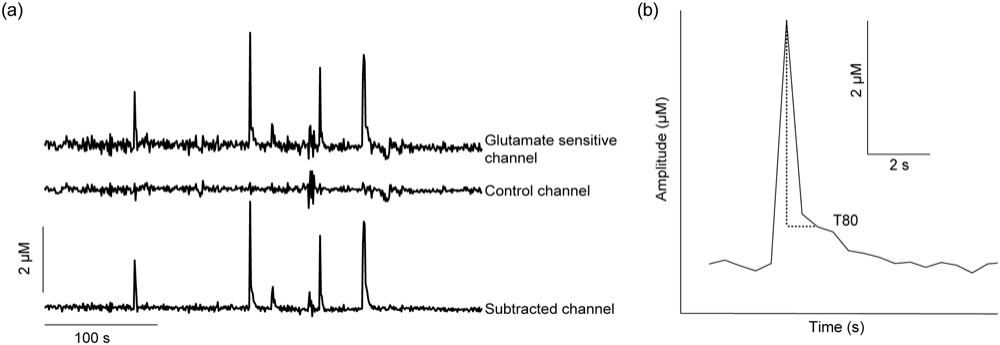
Recordings from a freely moving animal. a) A representative trace showing spontaneous glutamate transients in an animal postnatal day 34, within the third hour post-ethanol injection. b) Representative picture of a single glutamate transient from the subtracted channel. Amplitude (μM) is represented by the vertical axis and time in seconds on horizontal axis. T80 represents the time in seconds from maximum peak rise to 80% decay of signal (a measure of glutamate clearance).

**Fig 7:**
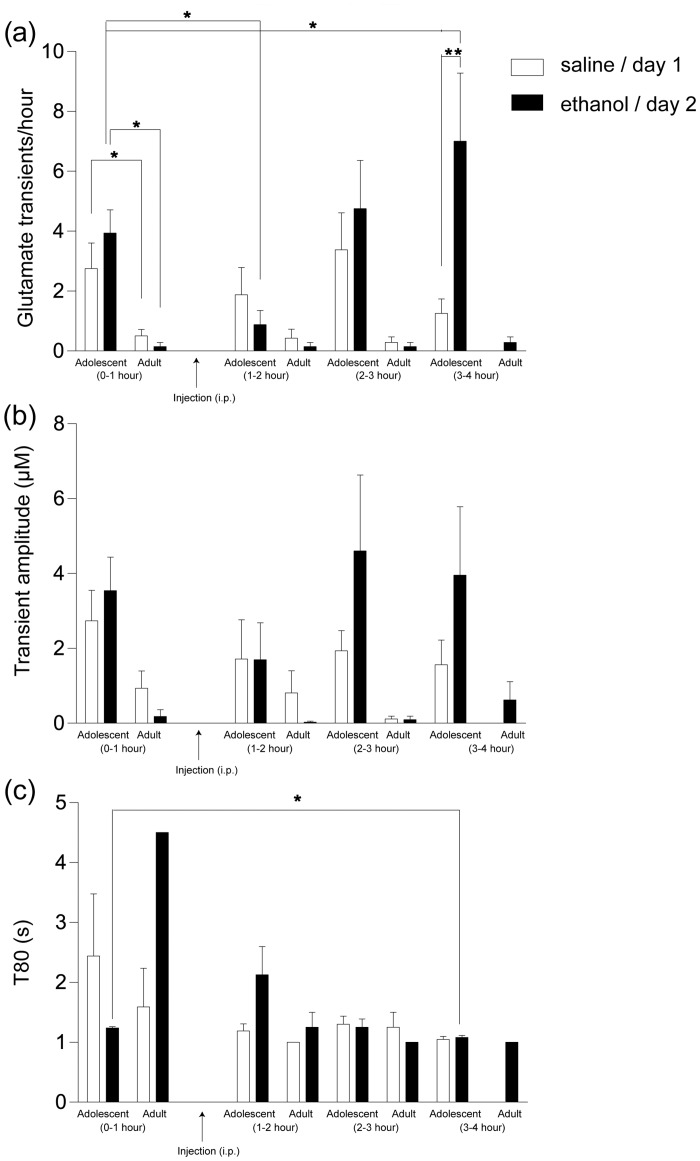
Effects of saline and ethanol injections on spontaneous glutamate transients in adolescent and adult animals. Experimental recordings were performed over two days: Experimental day 1 (saline injection, 6 ml/kg, unfilled bars) and experimental day 2 (ethanol injection, 1 g/kg, filled bars). Baseline recordings are represented as 0–1 hour on the *x* axis. Following baseline recordings, the animals received an intraperitoneal (i.p.) injection and recordings were continued for three hours. Every hour post injection is represented as 1–2 (first hour), 2–3 (second hour) and 3–4 (third hour) hour on the *x* axis. a) Experimental day 1 baseline recordings show that the transient frequency was higher in adolescent when compared with transients of adult animals (*p<0.05). The transient frequency was unaffected in any hour in both age groups post-saline injection compared to their respective baseline values. On experimental day 2, the transient frequency was higher in adolescent compared to adult animals (*p<0.05) during baseline recordings. An ethanol injection inhibited the transient frequency (*p<0.05) in the first hour and potentiated it in the third hour compared to the baseline values (*p<0.05) in adolescent animals. b) The averaged transient amplitude was higher in adolescent when compared to adult animals on both experimental day 1 and 2 during baseline recordings (p = 0.055 and p = 0.06, respectively). Averaged transient amplitudes were unaffected following saline injection in both age groups (p>0.05). Post-ethanol injection in adolescent animals, the average amplitudes decreased within the first hour and increased in the third hour. However, these changes in amplitude were not significant when compared to baseline values (p>0.05). In the adult animals, the average amplitude was unaffected following ethanol injection (p>0.05). c) We did not observe any significant difference between the averaged T80 value during baseline recordings of adolescent and adult animals (p>0.05). In adolescent animals, post-ethanol injection, in the third hour, the averaged T80 value decreased significantly (*p<0.05) when compared to the baseline values. Due to a low number of transients in adult animals, we could not perform any statistical analysis using T80 values. Also, note that the values missing or bars without SEM are due to the fact that the transient were absent in certain hours or, very low in frequency, respectively. All statistical comparisons were made using two-way ANOVA with Bonferroni´s comparison test and paired student´s t-test was used for analysis of T80. *p<0.05, **p<0.01.

Throughout the recordings on both experimental days, we observed spontaneous glutamate transients (Fig 6a and 6b). In the analysis of glutamate transients, we pooled the PND 28–38 (n = 5) and PND 44–55 (n = 3) animals together as adolescents (there was no significant difference in baseline frequency between animals PND 28–38 and PND 44–55, t = 1.83, p = 0.11) and compared them to adult animals (Fig 7). On experimental day 1, the frequency of glutamate transients during baseline recording was 2–4/hour in adolescent animals (n = 8) while the transient frequency was 0–1/hour in adult animals (n = 7, Fig 7a). A two-way ANOVA revealed an effect of age (F_1,13_ = 5.39, p<0.05) but no effect saline injection (F_3,39_ = 3.42, p>0.05) and no interaction effect over time (F_3,39_ = 2.09, p>0.05). Post-hoc analysis using Bonferroni’s multiple comparisons test showed no significant change in transient frequency of either adolescent or adult animals following saline injection. Average transient amplitude (Fig 7b) was 3.1 ± 0.9 μM (n = 8) in adolescent animals and 1.3 ± 0.5 μM (n = 7) in adults (F_(age)1,13_ = 4.43, p = 0.055). Transient amplitude was unaffected following saline injection in any age group (F_3,39_ = 1.91, p>0.05) and no interaction effect was found (F_3,39_ = 0.43, p>0.05).

On experimental day 2, the transient frequency was 3–5/hour in adolescent (n = 8) and, 0–1/hour in adult animals (n = 7) (F_1,12_ = 8.52, p<0.05, see Fig 7a). A two-way ANOVA revealed significant effects of ethanol injection (F_3,36_ = 4.44, p<0.01) and a significant interaction effect (F_3,36_ = 4.02, p<0.05). Post- hoc analysis using Bonferroni’s test showed that ethanol decreased transient frequency during the first hour following injection to 0#x2013;1/hour (p<0.05, see Fig 7a) and there was a rebound response during the third hour, the frequency increased to 7/hour (p<0.05, see Fig 7a) in adolescent animals. Moreover, when comparing transient frequency post injection (saline vs ethanol) in adolescent animals, we observed no significant difference between the first hour and second hour post-injection (p>0.05, saline vs ethanol). However, when comparing the third hour post-injection, we observed a significant difference in transient frequency (p<0.01, saline vs ethanol, as revealed by two-way ANOVA followed by a post-hoc analysis, Fig 7a). In adult animals there was no significant effect of any injection (saline or ethanol).

The amplitude of the spontaneous transients of adolescent animals (4.0 ± 0.8 μM, n = 8) was higher compared to that of adults (0.3 ± 0.2 μM, n = 7), but statistical significance was not reached (F_1,12_ = 4.04, p = 0.06, Fig 7b). Furthermore, the two-way ANOVA with post-hoc analysis using Bonferroni´s test revealed that ethanol had no effect on transient amplitude in either adolescent or adult animals (F_3,36_ = 2.21, p>0.05) and there was no interaction (F_3,36_ = 1.39, p>0.05, Fig 7b).

### Ethanol affects glutamate clearance in adolescent animals

To further analyze the dynamics of spontaneous glutamate transients in the mPFC we compared the clearance rate of transients in adolescent and adult rats. Clearance rate was termed as T80 and defined as the time point at which 80% of the peak amplitude has been cleared (Fig 6b). On experimental day 1, average T 80 values observed at baseline were 2.5 ± 1s (n = 8) for adolescent and 1.6 ± 0.6s, (n = 4) for adults, respectively (note that only 4 adult animals out of 7 showed transients during basal conditions, Fig 7c). Using two-tailed Student’s t-test for the above unpaired values we did not see any significant effect of age on the clearance rate of glutamate (p>0.5, t_9_ = 0.6). Using data from experimental day 1 we examined the effects of saline injection on T80 values in adolescent rats, no significant effects were observed (t_5_ = 1.24, p>0.05). In contrast, ethanol injection on experimental day 2 produced a significant attenuation in T80 during the third hour recordings (1.08 seconds ± 0.03, t_5_ = 3.70, p<0.05, n = 6) when compared to baseline T80 (1.24 ± 0.02 s). We used two-tailed Student’s t-test for paired values to test for significance. It is to be noted that 2 out of 8 adolescent animals had complete inhibition of transients throughout the three hours of recording following ethanol injection and hence we use an n = 6 of adolescent animals for T80 comparisons. Unfortunately, as the transient frequency in adults was already low during baseline conditions and, did not change post-injection, we do not have enough data points to make any statistical comparisons of T80 values before and after injection on day 1 or day 2.

## Discussion

In the present study, we observed an age-dependent decrease in basal glutamate levels in the mPFC of rats. PND 28–38 rats had higher basal levels than PND 44–55 which in turn, had higher levels than adult rats. Adolescent rats (PND 28–38 and PND 44–55) showed higher frequency of spontaneous glutamate release (transients) than adults. Moreover, in adolescent animals, we observed an initial inhibition in transient frequency followed by a delayed rebound increase of transient frequency following ethanol injection. In adult rats an acute ethanol injection did not induce any significant changes either in frequency or amplitude of glutamate transients.

To our knowledge, we are the first group reporting basal glutamate levels in the mPFC of adolescent rats. Interestingly, we here show that cortical glutamate levels decreased significantly with age. The glutamate levels in the mPFC of adult rats observed in this study is slightly lower than the concentration we have previously reported [19, 20]. This difference is probably a result of the fact that in our previous studies rats weighing 250–450 g were included i.e. higher glutamate levels from some adolescent rats (cut-off in the present study was PND 55, corresponding to an average body weight of 260 g in our set of rats) may have increased the average somewhat. One interpretation of the decreased basal glutamate levels at higher age would be that glutamate reuptake becomes more efficient as the expression of the major glutamate transporter, GLT-1, increases with age [26]. Although, there was a trend in this direction, changes in reuptake is probably not the main mechanism underlying the age-difference since we did not observe any significant difference in T80 values between adolescent and adult rats.

As studies using electrophysiology in brain slices have shown that acute ethanol may inhibit glutamate release in many brain regions [13–16] we expected to see a decrease in basal glutamate levels after an acute ethanol injection. However, ethanol (1 g/kg) did not significantly affect basal glutamate levels in either age group during our experiments. This result is in line with a microdialysis study, showing that acute ethanol injections at doses ranging from mild to heavily intoxicating (0.5 to 2.0 g/kg) did not affect glutamate output in the prefrontal cortex [10]. In contrast we observed an inhibitory effect of ethanol on spontaneous glutamate transients in adolescent rats. Spontaneous glutamate transients are short lasting peaks of glutamate that occur during recordings with MEA at a variable frequency and amplitude. Glutamate transients have previously been observed in the prefrontal cortex [27] and basolateral amygdala [28]. Herein we report a lower transient frequency than both previous studies which in addition to differences between brain regions may also be related to the fact that we introduced R^2^ correction and peak threshold. Moreover, we are using a recording rate of 2 Hz, which is considerably lower than that used in the study by Wassum and colleagues [28] at 80 KHz. A possible consequence of this sampling rate would be that transients with duration shorter than 500 milliseconds would not be detected. Transient amplitudes observed during our experiments are comparable with those reported previously. However, the clearance time (T80) observed by Hascup and colleagues [27] varied considerably more than those observed by us and Wassum and colleagues [28]. There is a possibility that glutamate transients may vary depending on age, strain of animals, brain region etc. Also, methods used for analyzing transients may have to be optimized and standardized before we can directly compare transients recorded in different experiments.

Functionally, glutamate transients in basal amygdala were found to precede lever-pressing behavior to obtain sucrose reward. The transients recorded had no effect on the basal levels of glutamate. Interestingly, infusion of the potent sodium blocker tetrodotoxin into the basal amygdala of these rats resulted in a decrease in transient frequency as well as a complete blockade of the behavior [28]. Apparently, spontaneous glutamate transients, rather than changes in basal levels of glutamate, may better reflect rapid glutamatergic synaptic processes. Assuming that transients correlate with behavior, which seems plausible according to Wassum and colleagues [28], the fact that our experiments were performed during daytime in an enclosed box and that our rats were not behaviorally active for the most part based on gross examination, may be another contributing factor to the low transient frequency observed in our rats. Nevertheless, this allowed us to study specifically if and how ethanol affects glutamate dynamics in the mPFC in the developing brain, which is basic knowledge necessary to understand and interpret data from more complex behavioral studies. It is highly likely that behavior *per se* changes glutamate transmission in the prefrontal cortex and in an experiment designed to study the pharmacological effects of ethanol behavior would probably complicate data interpretation.

Our result showing that acute ethanol injection initially inhibited glutamate transients in adolescent animals is in line with previous data from electrophysiological recordings, suggesting that ethanol inhibits glutamate release in pre-adolescent and adolescent animals, possibly via inhibition of L-type calcium-channels on presynaptic terminals [29] and/or via presynaptic gamma-aminobutyric acid-B receptors [14]. As mentioned in the introduction, ethanol has been found to decrease firing rate of mPFC presumed pyramidal neurons in anesthetized animals [11]. In a follow-up study, Weitlauf and Woodward (2008) [12] demonstrated in a slice preparation that ethanol inhibits the spontaneous firing rate via blockade of postsynaptic NMDA receptors without any effect on presynaptic glutamate release. As we observe a reduction of transient frequency as well as a rebound increase following ethanol our data suggest that in addition to or as a consequence of postsynaptic NMDA receptor inhibition ethanol may affect activity of pyramidal neurons by changing afferent glutamatergic input.

The mechanism by which ethanol modulates glutamate transients remains to be determined. We did, however, observe a change in glutamate clearance that may represent a physiological mechanism or a homeostatic response of the brain trying to maintain optimal glutamate levels. An increase in transient frequency during the third hour following ethanol injection was associated with a tendency to faster clearance during the recovery phase indicating dynamic adaptations in glutamate transporter activity. Basal levels of glutamate have been suggested to be tightly regulated mainly by the Na^+^-dependent glial glutamate transporters GLAST and GLT-1 [30, 31]. The effect of acute ethanol on these specific transporters is currently unknown and chronic treatment with ethanol does not seem to affect the expression levels of these transporters in the brain [32]. However, in cortical astrocyte cultures, acute ethanol exposure was shown to inhibit glutamate uptake, via reduced protein kinase C-mediated phosphorylation of glutamate transporters [33]. Thus, the effect on reuptake of glutamate observed in our experiments may be related to an interaction between ethanol and glutamate transporters.

In summary, the herein observed difference in basal glutamate levels as well as glutamate transients between adolescent and adult animals support the notion that significant changes in cortical glutamatergic neurotransmission occur during development. The age-dependent difference in sensitivity to ethanol reported herein is in line with several previous studies showing that acute ethanol affects glutamatergic transmission to a higher degree in adolescent animals than in adults [16, 34–36]. The effects of ethanol exposure on prefrontal glutamate dynamics may disturb the maturation of the developing adolescent prefrontal cortex and with this, inhibit the development of normal cortical functions such as behavioral control over risk-taking and ethanol over-consumption.
